# The future of U.S. social prescribing: foundations, implementation, and leadership summit insights

**DOI:** 10.3389/fpubh.2026.1789606

**Published:** 2026-06-04

**Authors:** Alan Siegel, Barry Zuckerman, Ardeshir Z. Hashmi, Serena Shapard

**Affiliations:** 1 UCSF Medical Center, San Francisco, CA, United States; 2 Boston University School of Medicine, Boston, MA, United States; 3 Cleveland Clinic Center for Geriatric Medicine, Cleveland, OH, United States; 4 Bryn Mawr College, Bryn Mawr, PA, United States

**Keywords:** aging, arts prescribing, nature prescribing, social connection, social drivers of health, social health, social prescribing

## Abstract

The U.S. healthcare system is confronting at least three converging crises: a rapidly aging population with complex needs, rising prevalence of mental illness, and an epidemic of social isolation that contributes substantially to morbidity, mortality, and healthcare costs. Current clinical approaches cannot meet demand due to persistent workforce shortages, limited treatment capacity, and variable patient adherence. Social prescribing (SP), which systematically connects patients from healthcare settings to community-based non-clinical supports, including social, cultural, physical activity, and nature-based programs, has been developed in over 30 countries and is emerging in the United States as a promising adjunct to clinical care. This paper synthesizes epidemiologic, economic, and implementation evidence to examine the role of SP in addressing these strains on domestic healthcare infrastructure. Drawing on data from a national survey of SP movement leaders ahead of the 2025 U.S. SP Leadership Summit, we identify key barriers and opportunities across patients, clinicians, community partners, and health systems. Major challenges include limited awareness, fragmented referral pathways, measurement deficits, and a complex multi-payer environment. Despite these barriers, substantial opportunities exist to align SP with U.S. public health priorities, including mental health prevention, health equity, social drivers of health, lifestyle medicine, and value-based care. We argue that SP represents a low-risk and high-value strategy to improve wellbeing and reduce healthcare burden by formally recognizing the importance of social connection to health outcomes. Strategic investment in policy frameworks, sustainable financing, workforce development, technology, and research is essential to supporting system-wide adoption of SP in the United States.

## Introduction

U.S. healthcare systems are under mounting strain, threatening health and economic outcomes. Large-scale change is urgent, and models that extend beyond symptom treatment to address prevention and the social drivers of health (SDOH) hold promise for attenuating these stressors.

Social prescribing (SP), which involves structured referral of patients from healthcare settings to community-based, non-clinical services, is one such approach. As SP programs expand globally, interest in adapting this model to the United States grows. Understanding domestic barriers and opportunities is necessary to design feasible and responsive SP approaches in the U.S. context.

Importantly, SP differs from informal lifestyle advice. Rather than simply encouraging patients to improve their nutrition, exercise, or social networks, SP involves documented, structured referrals to community-based programs, often related to the arts, physical activity, time in nature, social connection, and volunteering, each with established links to improved health outcomes. These referrals are typically facilitated by a trained intermediary, often called a *link worker*, who assesses patients’ needs and guides navigation to appropriate resources. By formalizing pathways to non-clinical supports and enabling follow-up and outcome tracking, SP provides a framework for addressing SDOH, including social connection, that are not always adequately managed through clinical care alone.

The urgency of exploring SP in the United States is made clear through the following trends:

### An aging population

Demographic change is a central driver of urgency. Currently, 18% of the U.S. population is over 65 years of age, a proportion projected to exceed 71 million by 2030 (1). Older adults account for 37% of personal healthcare expenditures (2), largely due to higher rates of chronic disease. More broadly, individuals with chronic and mental health conditions account for 90% of the nation’s $4.9 trillion in annual healthcare spending (3). Because the prevalence of these conditions increases with age, population aging is expected to amplify both clinical demand and financial pressure.

### Rising rates of mental illness

Mental health needs are also rising while access to care remains limited. An estimated 23.2% of U.S. adults experience some form of mental illness (4), including 13.1% with major depressive disorder (5) and 12.1% with anxiety disorders (6). Of the 59.3 million adults living with mental illness, only about half receive any form of treatment (7). Workforce shortages exacerbate these gaps. Approximately 47% of the U.S. population resides in a Mental Health Professional Shortage Area (8), and deficits in the behavioral health workforce are projected to persist into the next decade (9). Although psychotherapy and pharmacotherapy remain foundational treatments, they are not accessible or sufficient for all individuals. Integrating community-based programming through SP offers a complementary strategy that can support individuals with mild to moderate mental health concerns, particularly anxiety, depression, and loneliness, reducing strain on healthcare systems and enabling providers to focus on the most severe cases.

### An epidemic of loneliness and social isolation

Loneliness and social isolation constitute an additional and historically under-addressed public health concern. Approximately one-third of U.S. adults report experiencing loneliness, and one-quarter experience social isolation (10). Both are associated with substantial morbidity and mortality, including an estimated 30% increase in earlier death (11), elevated risks of cardiovascular disease and stroke (12), and increased risk of cognitive decline and dementia (13). These social conditions also carry significant economic consequences; loneliness costs $407 billion annually to the US economy, with $6.7 billion annually borne by Medicare due to social isolation, with additional costs borne by Medicare due to social isolation (14). Reflecting the growing recognition of these impacts, the World Health Organization’s 2025 report, From Loneliness to Social Connection, identifies SP as a key community-based strategy for strengthening social connection (15).

### Economic and social promise of social prescribing

Evidence from countries with established SP programs suggests potential system-level benefits. Reported returns on investment include approximately £2.80 for every £1 spent in the United Kingdom (16) and $4.43 for every $1 invested in Canada (17). Evaluations have also documented reductions in healthcare utilization, including fewer emergency department visits, hospitalizations, and primary care appointments. In Australia, whose healthcare structure shares similarities with that of the United States, SP participation has been associated with a 17% reduction in hospitalizations and a 27% decline in overall health service use (18). While results vary across settings and study designs, these findings indicate that SP may help mitigate the clinical and financial pressures associated with aging, mental illness, and social disconnection.

In this context, SP warrants serious consideration as a component of U.S. healthcare reform. By formally linking patients, particularly those with high healthcare utilization, demonstrated loneliness/isolation, or symptoms that may be attenuated with improved social connection to group-oriented activities in the community, SP addresses upstream drivers of health alongside medical care. Continued research is needed to clarify optimal implementation models, workforce requirements, financing mechanisms, and long-term outcomes in the U.S. setting.

## Methods

This Perspective draws on peer-reviewed literature, policy reports, and implementation evaluations identified through targeted searches of major databases and review of key references.

We also conducted a descriptive analysis of a pre-event survey administered to attendees of the inaugural U.S. SP Leadership Summit in October 2025. The survey was designed to assess perceived barriers and opportunities related to SP implementation in the United States. Respondents (*n* = 28) include U.S. and international leaders in SP from healthcare, community organizations, research, and policy sectors. Survey findings were synthesized and organized by stakeholders and by theme (see Supplementary Appendix).

## Results

### Barriers

Barriers were notably categorized by audience and by systematic component, highlighting the importance of designing SP programs that clearly identify the intended audience and address specific priorities accordingly.

### Audience barriers

#### Patients

Patients may be entirely unaware of SP as an option (19) or of SP “destination” resources in their community. They may be hesitant to engage in discussion regarding SP, secondary to stigma associated with social isolation and loneliness (20), and worry about not being included or affordability. While the Centers for Medicare and Medicaid Services is increasingly encouraging health systems to move toward value-based care (21), much of the health system remains entrenched in a fee-for-service payment model. This results in patients experiencing time-constrained visits with their clinicians, with discussions regarding SP perennially deferred.

#### Clinicians

Under financial and time pressure, clinicians grapple with addressing competing medical conditions, associated quality metrics, and the need to meet patients’ needs and preferences. While many doctors recommend that patients join a gym, exercise more, or eat better, most are unaware of SP as a specific recommendation with a framework of referrals to community organizations (22). There is also a lack of consensus on measurement tools to identify social isolation, patient priorities, or tracking the impact of SP (23). Limited clinician referrals to community organizations and patient willingness to engage with them may also reflect a healthcare hierarchy that prioritizes a biomedical over a socio-ecological care philosophy (24).

#### Community partners

Community organizations as partners are integral to delivering SP. They may, however, face capacity constraints, reducing the community workforce’s availability for outreach, follow-up, or tracking. There is also a lack of patient advocacy for SP as a public health imperative.

Another limitation is the current absence of a consolidated, easily accessible, and trackable repository for SP destination resources. This results in fragmented, potentially redundant SP care pathways with high risk for loss to follow-up. Additionally, there is a scarcity of mechanisms to tailor SP to the vulnerable patient groups (e.g., LGBQT, older adults, non-English speakers, patients with mental health challenges, disability, multimorbidity, and substance use disorders) (25).

### Systemic barriers

Systemic barriers to implementation of social prescribing include infrastructure, evidence, awareness, educational, and financial barriers.

#### Infrastructure

The lack of a unified national framework (such as the United Kingdom’s National Health Service) and a federal and state SP policy vacuum make integration across siloed health systems and community organizations challenging. This is compounded by a lack of financial incentives and regulatory support for SP implementation. There is limited health system buy-in due to the lack of a clear U.S. SP return on investment and competing organizational priorities amid increasing financial and regulatory pressures. This, in turn, inhibits health systems’ support for scaling SP.

The U.S. lacks a clear model or reimbursement mechanism to support the critical link worker role, unlike the U.K., where the National Health Service supported 3,500 link workers in 2024 (26).

Access to SP prescribers and community offerings is challenging. Lack of appointments, language translation, and transportation availability, scheduling navigation support, and necessary accommodations for sensory and physical impairments are all contributory.

Fragmentation of care abounds, with poor communication between primary care, specialty care, and community health organizations, and no unified care plan visible to all. Limitations in electronic health record interoperability are emblematic of the same problems. This results in healthcare system and community partner silos.

#### Evidence gaps

Research on U.S.-based SP outcomes, whether health-related, implementation, feasibility, or fiscal, is lacking, with the ensuing misperception of SP as a “soft science.” There is a strong need to build a robust evidence base to elucidate what works, for whom, and why, including cost–benefit analysis. There is a lack of standardization of SP best practices, protocols, quality control, and outcome measures. While there is a good deal of research from other countries on SP, many feel these studies need to be replicated in the U.S., as our health system differs significantly from those in other countries.

#### Educational and awareness needs

There is a need for a standardized, high-quality SP training, certification, and education curriculum for SP professionals in both the healthcare and community organization settings. There must also be efforts in medical school and residency education, allied health professional schools, and public-facing educational programs. Education is also needed for frontline community “SP champions” (i.e., in religious and community centers, libraries, schools, and community colleges).

Public health awareness campaigns regarding SP, such as those by the U.S. Surgeon General (27), are needed to promote cross-sector dissemination. Media coverage that uses “solution journalism,” resulting in books such as “The Connection Cure” (28), and coverage in the popular press will help immensely in this regard. This public awareness often takes a long time. In the U.K., where SP has been scaled nationally since 2019, a 2023 study found that only 1/3 of the public knew about SP (22).

#### Financial

The U.S payer landscape is complex. Unlike single-payer systems in the United Kingdom or Canada, the payor spectrum in the United States spans Medicare, Medicaid, private for-profit and not-for-profit models, philanthropy, etc. An insurer/payor misalignment currently exists due to a lack of insurer incentives to cover SP care and reimburse SP providers. A business case for SP must therefore be made for fee-for-service, value-based payment, and philanthropic donor contexts.

Even if funding for SP is secured, sustainable long-term funding for SP programming remains lacking. Funding is often delayed as programs await funding negotiations (29), and time frames are often too short for operationalization (30). SP pilot programs often die in the “valley of death” (31) once initial “seed” funding ends. This may be secondary to a lack of SP awareness amongst payors, investors, and philanthropists. The potential cost savings would be a strong incentive to fund this work more widely, and several health insurers are beginning to recognize this potential.

### Opportunities

#### Building on success and awareness

Aligning with social and public health priorities is a great opportunity in SP. The U.S. population has developed an appreciation of loneliness and social isolation, and the negative effects on our health. This is an area that has not traditionally been addressed within U.S. healthcare. SP is positioned to help address the epidemic of loneliness by utilizing the health system and promoting community connection and family resilience.

Regarding mental health, SP can help with youth and older adult prevention, resilience building, and integration with mindfulness/positive psychology approaches. For addressing health equity and the social drivers of health (SDOH), there are early efforts for SP to collaborate with the SDOH research sector. In order to increase SP at Kaiser Permanente, it is being incorporated into their ongoing Social Health (SDOH) efforts. SP should be added as a mechanism more widely to address social drivers of health and reduce inequities. The area of Food as Medicine has already received significant attention and resources, and this would be a good area for collaboration with SP efforts.

The arts in public health and arts prescribing movement has already established significant momentum and a strong research basis in the U.S. Large efforts like One Nation, One Project, which highlighted arts engagement in 22 cities across the country on July 27, 2024, strengthened partnerships between arts, health, and local governments (with groups such as the National League of Cities) with the intent of improving community trust, engagement, and health outcomes through participatory arts (32). Social Rx (Previously Art Pharmacy) has been developing its business model at numerous locations across the country, including with Stanford undergraduates and the Massachusetts Cultural Council. Late in 2024, a report of 23 case studies of a combination of arts and other SP modalities was released (33). There is great activity in arts in health and arts prescribing research among the Johns Hopkins NeuroArts Lab, the EpiArts Lab at the University of Florida, and the WHO Jameel Arts and Health Lab. Recent books have synthesized the research and found that the arts have clear and impressive benefits for mental health, loneliness and social isolation, and chronic disease (34, 35).

## Discussion

SP incorporated into the health system has significant potential to reduce costs. Examples include showing how Medicare, Medicaid, or private insurance could reduce costs and improve outcomes. In looking at value-based care, there are opportunities to engage payers, large employers, and purchasers of care. A strong business case will be key to SP adoption.

There are great opportunities for partnership and ecosystem building. In the healthcare and wellness realm, partnerships could be formed with hospitals, health plans, community centers, food pantries, and organizations such as the YMCA. There is also the opportunity to develop cultural partners and connect with arts institutions, museums, and arts/health collaboratives. Present examples of these include the partnerships that Art Pharmacy has developed with many arts organizations. The Johns Hopkins NeuroArts lab, via the International Arts + Mind Lab, has also developed collaborations with arts organizations.

Another potential area of collaboration is with the nature and nature prescribing sectors. There is strong evidence that being out in nature improves physical, mental, and cognitive health across all diverse populations (36). There is an ever-increasing presence in the media about these health benefits, including from the National Park Service, in 2025 (37).

In addressing the individual’s well-being, other movements naturally overlap with SP. One such example is the $500 billion U.S. wellness economy, which is aligned with Gen Z and millennial priorities. Relatedly, there is also a natural connection to the work in Lifestyle Medicine, which is also gathering momentum in the U.S. and has been partnering with Blue Zones LLC to accelerate a lifestyle-first approach across health systems (38). There is also potential crossover with the stated objectives of the Make America Health Again strategy, with a concentration on lifestyle, prevention, and social connection.

In the areas of policy, funding, and sustainability, work is needed to develop SP reimbursement models with insurance companies, establish clear payment models, and push health plans to include SP. Policy frameworks need to be designed that demonstrate the need for state and federal incentives, policy support, and sustainable funding for link workers, community navigators, and community partners.

Efforts need to be made to advance technology and digital pathways, including platforms that connect providers with community resources. Telehealth could expand SP practices into virtual formats, especially for rural/underserved areas. In addition, digital referral and monitoring systems are needed widely to measure outcomes and improve personalization.

There is a clear need for a national advocacy organization to help establish best practices, assist with training development, and collaborate and convene with the many sectors that comprise SP. Social Prescribing USA has played that role and is now beginning the transition from an all-volunteer organization to a professionalized one that could also provide training, support adoption of SP, and set the research agenda.

While many pilots are running across the country, successful pilots need to be brought to scale, moving beyond non-standardized pilots to standardized, system-wide approaches. In Kaiser Permanente in Oakland, the SP pilot is being built into Social Health (SDOH) to enable scalability once the pilot is complete.

## Conclusion

While the implementation challenges are real, the opportunity to improve patient health and wellbeing through SP is profound. Especially for high-risk or high-utilizing patients, this is an idea who time has come. The multiplied costs of treating an increasingly aging population with high rates of loneliness, social isolation, and mental illness in an already stressed healthcare system are not sustainable in our present models, as increasing morbidity is already growing.

One approach to spreading SP could be for clinicians to start offering a simplified version to a small number of patients. SP, led by clinicians who bring their insight and creativity to develop innovations in care delivery, can be incorporated and grow in a much shorter time frame and at lower cost than our traditional approach, which often takes 10–20 years. By building on interventions with known value and acceptance (e.g., movement as a treatment for depression), utilizing solid research findings from outside the U.S., and doing small trials of change (aka Lean Startup) (39), innovations could be adapted to serve our patients with low-risk, low-cost interventions. An educational program to support clinician education and implementation in a Lean Startup model is now being developed as part of the Kaiser Permanente pilot.

There are many approaches available to grow SP in the U.S. It is likely that various approaches will be tested in different health centers and health insurers across the country as our own best practices for SP are developed. Given the challenges we face in the U.S., it is clear that we need to adopt SP as a critical part of the solution. We simply cannot afford to continue business as usual.

## Data Availability

The original contributions presented in the study are included in the article/Supplementary material, further inquiries can be directed to the corresponding author.
