# Supplementary material for: The future of U.S. social prescribing: foundations, implementation, and leadership summit insights
Source: Front Public Health. 2026 Jun 4;14:1789606. doi: 10.3389/fpubh.2026.1789606 (PMC13275706; doi:10.3389/fpubh.2026.1789606)
Supplement: Supplementary file 1 [file Data_Sheet_1.docx]

**Supplementary Appendix**

The following tables summarize the responses of 28 attendees of the inaugural US SP Leadership Summit (in October 2025), to a pre-event survey designed to understand perceived barriers and opportunities for SP in the United States. Respondents are leaders of SP in the US and globally and were invited to the Summit in recognition of their significant contributions to the field of SP. They represented most sectors involved in SP, including health professionals, health insurers, arts-in-health professionals, nature prescribers, exercise specialists, educators, community-based organization leaders, researchers, and funders.

**Supplementary Table S1**

**What are the biggest barriers to the success of SP in the United States?**

*Responses could similarly be grouped into six major themes: economic, evidence, messaging, infrastructure, clinical workflow, and community capacity*

| *Theme* | *Sub-theme* | *Description* |
| --- | --- | --- |
| Economic | Insurance | Lack of coverage and absence of reimbursement models |
|  | Value-based care gap | Limited value-based contracts and incentives |
|  | ROI concerns | Little cost-benefit data available; existing data is of mixed quality |
|  | Funding | Reliance on pilots without guaranteed sustainability  Insufficient funding sources for long-term growth |
| Evidence | Research gaps | Lack of robust, credible evidence showing sustained outcomes and cost savings |
|  | Soft science perception | Seen as less rigorous as compared to biomedical approaches |
| Messaging | Low familiarity | True among patients, families, practitioners, funders, policymakers, and community-based organizations |
|  | Cultural values | US values often prioritize work/productivity over social connection |
|  | Perception | SP sometimes perceived as “elite” or inaccessible |
|  | Buy-in difficulties | Limited organizational acceptance and/or resistance within healthcare settings |
| Healthcare infrastructure | Fragmentation | US healthcare is siloed; no national framework like the UK |
|  | Policy vacuum | Lack of coordinated state/federal policy support |
|  | Competing priorities | Short-term cost control often outweighs innovation adoption |
|  | Infrastructure gaps | Weak systems, insufficient integration capacity |
| Clinical workflow | Time constraints | Limited time during patient visits  “Clicker fatigue” |
|  | Habits | Clinicians sometimes resistant to new practices once accustomed to existing methods |
| Community capacity | Resource limitations | Community-based organizations often under-resourced to handle referrals |
|  | Integration challenges | Difficult to align local organizations with large healthcare institutions |

**Supplementary Table S2**

**What are the biggest opportunities for SP in the United States?**

*Responses could be grouped into six major themes: partnerships, messaging, technology, evidence, policy, and education*

| *Theme* | *Sub-theme* | *Description* |
| --- | --- | --- |
| Partnerships | Health system integration | Partnership with Medicare and Medicaid plans to reduce costs and increase accessibility and promotion of value-based care  Advocacy with insurance companies and collaboration to develop clear payment models |
|  | Community partnerships | Collaboration with community centers, food pantries, and “third places” like YMCAs.  Collaboration with arts institutions, museums, state/national parks, and art & health initiatives (e.g. Art Pharmacy) |
|  | Educational partnerships | Collaboration with the American College of Lifestyle Medicine |
| Messaging | Social & Public Health Priorities | Addressing the “epidemic of loneliness” and promoting community connection and family resilience.  Using SP to address social determinants of health (SDoH) and reducing inequities  Food as Medicine: Nutrition and food access as a social prescription category |
|  | Wellness market | Positioning SP within the $500B wellness economy in the United States, aligned with Gen Z and millennial priorities |
| Technology | Virtual SP | Expanding SP into virtual formats, particularly for rural and underserved areas |
|  | Prescription databases | Tools to connect providers with community resources |
|  | Digital data collection | Digital referral and monitoring systems to measure outcomes and improve personalization |
| Evidence | Chronic illness management | Demonstrating and documenting the widespread applications of SP beyond mental illness treatment, including management of chronic pain, cardiovascular, and neurologic conditions |
|  | Economic impact | Documenting cost savings of short- and long-term SP interventions |
|  | Evidence accessibility | Publishing in high-impact journals and developing an accessible library of SP evidence |
|  | Systematic pilots | Moving from non-standardized to standardized, system-wide approaches to pilot SP interventions |
| Policy | Incentives | Establishment of state/federal incentives for SP |
|  | Link worker support | Establishing reliable funding for link workers, navigators, and community partners |
| Education | Professional education | Developing curricula, training programs, and certifications for SP professionals |
|  | Student education | Integration of SP into college and medical school curricula |
|  | Public education | Public-facing educational campaigns about SP |

**Supplementary Table S3**

**What are the greatest opportunities for academic research on SP in the United States?**

*Five high-priority areas were health outcomes, economic impact, patient/provider engagement, metrics development and policy/systems research*

| *Topic* | *Sub-topic* | *Description* |
| --- | --- | --- |
| Health outcomes | Clinical impact | Effects on chronic disease management, mental health, social isolation, loneliness, anxiety, depression |
|  | Population-specific research | Outcomes for priority groups like older adults, veterans, children/youth, people with chronic illness, and rural/underserved populations |
|  | Program effectiveness | Which protocols work best (type of activity, frequency, dosage of engagement) |
| Economic impact | Cost effectiveness | Do SP programs reduce ED visits, hospitalizations, and medication use? |
|  | ROI | Savings per dollar spent for payers, providers, states, and employers |
|  | Value-based care impact | Long-term sustainability and potential |
|  | Fiscal models | How to structure funding and reimbursement for the US context |
| Engagement | Patient engagement | What motivates patients to participate and stick with a prescription?  How does patient-provider collaboration impact uptake and outcomes? |
|  | Provider behavior | What motivates providers to prescribe, and which types of providers are most likely to do so? |
|  | Volunteer engagement | How are volunteers retained for assisting with prescriptions? |
| Measurement standardization | Metrics development | Development of validated, standardized outcome measures |
|  | Domains of measurement | Wellbeing, loneliness, social connection, community engagement, quality of life |
|  | Interoperability | Systems for tracking and sharing data between healthcare and community organizations |
| Policy & systems | Governance models | Best structures for durable health-community partnerships |
|  | Mapping | Where SP programs currently exist across the US  Regional variations in outcomes |
